# Investigation of risk factors for external ventricular drainage‑associated central nervous system infections in patients undergoing neurosurgery

**DOI:** 10.3892/mi.2023.104

**Published:** 2023-08-29

**Authors:** Charalampos Gatos, George Fotakopoulos, Maria Chatzi, Vasiliki Epameinondas Georgakopoulou, Demetrios A. Spandidos, Demosthenes Makris, Kostas N. Fountas

**Affiliations:** 1 Department of Neurosurgery, General University Hospital of Larissa, 41221 Larissa, Greece; 2 Department of Critical Care Medicine, General University Hospital of Larissa, 41221 Larissa, Greece; 3 Department of Infectious Diseases and COVID-19 Unit, Laiko General Hospital, Medical School, National and Kapodistrian University of Athens, 11527 Athens, Greece; 4 Laboratory of Clinical Virology, School of Medicine, University of Crete, 71003 Heraklion, Greece

**Keywords:** ventriculitis, meningitis, external ventricular drain, central nervous system infections, neurosurgery

## Abstract

Meningitis/ventriculitis (MV) is an illness which can occur as a complication following neurosurgical procedures. Devices such as an external ventricular drain (EVD) are also related to considerable complications, such as infections. The present study examined the risk factors associated with central nervous system (CNS) infections associated with the external ventricle drainage system. The present retrospective study included all patients hospitalized between April, 2011 and August, 2018 who had been receiving therapy with EVD for developed hydrocephalus. A total of 48 out of 65 patients were classified into two groups as follows: Patients without MV (group A) and patients who developed MV (group B). The durations of hospital stay and intensive care unit (ICU) stay were significantly lower in group A (32.4±24 and 21.1±11 days, respectively) compared to group B (54.7±37 and 42±24 days, respectively) (P=0.027 and P=0.001, respectively). The Acute Physiological and Chronic Health Evaluation II (APACHE II) score and EVD distance from the wound exit side to the burr hole were significantly lower in the survivors compared to the non-survivors (17.5±6 and 15.4±4 vs. 22.5±6 and 39.8±38, respectively). Receiver operating characteristic analysis revealed that the APACHE II score with an area under the curve [(AUC) of 0.677, P=0.044, and 95% confidence interval (CI) of (0.516-0.839)] and a cut-off value of 14 could predict mortality with a sensitivity of 100% and a specificity of 71%; the EVD distance from the wound exit side from the burr hole with an AUC of 0.694 (P=0.028), 95% CI of 0.521-0.866 and a cut-off value of 11.5 mm could predict mortality with a sensitivity of 88% and a specificity of 83%. On the whole, the present study demonstrates that the EVD-related distance from the wound exit side of the burr hole can predict poor outcomes due to CNS infections in patients undergoing neurosurgery.

## Introduction

Meningitis/ventriculitis (MV) is a complication that can occur following neurosurgical procedures (1-4). Devices such as an external ventricular drain (EVD) and intracranial pressure (ICP) monitors are critical for the management of an elevated ICP, bleeding and monitoring in patients undergoing neurosurgery (5). However, the use of such devices is associated with considerable complications, including misplacement, bleeding and infections (2). The incidence of infection among patients in which an EVD is used ranges between 0 and 22%, while multidrug resistant (MDR) and extensively drug resistant microorganisms have been reported (3). These infections are associated with high morbidity and mortality rates, they significantly prolong the duration of hospital stay, and thus also increase the costs and often negatively affect the overall prognosis of patients (3,6,7).

The present study aimed to examine the risk factors for central nervous system (CNS) infections associated with the use of the EVD system and to assess the effects of an infection control intervention targeting the improved management of cerebrospinal fluid (CSF) drains.

## Patients and methods

### Study design

The present retrospective study performed in a tertiary care academic neurosurgical center at the University Hospital of Larissa (Larissa, Greece). The study included all patients hospitalized between April, 2011 and August, 2018 at the neurosurgical and intensive care unit (ICU) who had been receiving therapy with EVD for developing hydrocephalus following different neurosurgical interventions. In total, 20 patients (41.7%) developed MV, and the cases that presented positive cultures or Gram-positive stains in the CSF, but normal levels of glucose, protein and cytology were considered to as contamination cases but not infection cases, and were thus excluded from the study. In the final pool, 48 patients out of the 65 patients were included and these patients were divided into two groups. Data collection was performed by two qualified research nurses (MC and CG), and the data were reviewed and analyzed by two physicians (GF and VEG) on the basis of a structured form that included questions related to protocol. The protocol included points (listed and defined statistically) related to a list of variables (items, ‘database fields’) that were essential for the study to be completed, such as the characteristics of the population (i.e., age, sex, medical history), type of treatment, microbiology (including bacterial susceptibility to antibiotics), hospital-related infections, and the main end points (overall survival, adverse effects related to treatment). The Institutional Review Board (IRB) of University of Thessaly, Greece/The School of Medicine/School of Health Sciences approved the study (IRB no. 28122/18-05-2018). The present study was in line with the Declaration of Helsinki (1995; as revised in Edinburgh 2000). Written informed was obtained from all the included patients.

Following inclusion, the patients were classified into two groups: The patients without MV (group A; n=28) and patients who developed MV (group B; n=20).

### Study endpoints

The primary outcome was in-hospital mortality. Secondarily, the length of hospital stay, ICU stay and the Glasgow Coma Scale were examined in patients who were discharged alive (GCSexit).

### Clinical data and definitions

The severity of the clinical condition according to the Acute Physiological and Chronic Health Evaluation II (APACHE II) scoring system was retrieved from records of the patients and was then documented. The APACHE II score is a severity-of-disease classification system that is applied within a 24-h timeframe following the admission of a patient. An integer score from 0 to 71 is computed based on several measurements; higher scores correspond to disease which is more severe disease and to a higher risk of mortality (8). The findings from computed tomography (CT) brain scans were used to describe the cause of admission as follows: Traumatic brain injury, the presence or absence of subarachnoid hemorrhage at the basal cisterns and intraventicular involvement, intracerebral or/and intraventricular hemorrhage. Nosocomial MV was defined according to definition provide by the Centers for Disease Control and Prevention (CDC) (9).

Infections of the CNS are diagnosed according to at least one of the following two criteria: The presence of a microorganism isolated from CSF and a fever >38˚C in the absence of any other recognized cause, as well as any of the following: An increased number of leukocytes (>10,000 per mm^3^ with >50% polymorphonuclear leukocytes), increased protein (>45 mg/dl) and/or decreased glucose levels (40 mg/dl) in the CSF. Herein, patients were considered to have a mixed bacterial infection when two or more microorganisms were isolated from the CSF cultures. A positive culture or Gram-positive stain in the CSF with normal levels of glucose and protein and a normal number of cells in the absence of symptoms was not considered an infection. According to the institution's therapeutic protocol, patients were considered to have been cured if they presented no fever over the previous 10 days and no isolated microorganisms in the previous two CNS cultures (10).

In the present study, CSF samples were obtained with the use of an intraventricular catheter or lumbar puncture at rest. Gram-positive MDR bacteria were methicillin-resistant *Staphylococcus aureus* and *Enterococcus faecium*. Colistin-resistant bacteria were considered isolates with minimal inhibitory concentrations >2 µg/ml by both broth microdilution and E-test methods.

The morphology, Gram stain and reactions with the Vitek 2 GNI card (bioMe'rieuxVitek, Australia, Pty Ltd., Generic Network Interface) were all used to identify the microbial clinical isolates.

### Statistical analysis

Data are expressed as the mean ± SD. Data were assessed for normality using the Shapiro-Wilkes test. Nominal data were analyzed using the Fisher's exact test. Continuous data were analyzed using an unpaired Student's t-test or the Mann-Whitney U-test, as appropriate. The discriminative ability of significant variables was evaluated by using the area under the receiver operating characteristic curve (ROC) (AUC). A P-value <0.05 was considered to indicate a statistically significant difference. Statistical analyses were performed with the use of Statistical Product and Service Solutions (SPSS) software, version 15 (SPSS Inc.).

## Results

A total of 48 patients were hospitalized during the study period and were treated using an EVD. A total of 20 patients (41.7%) developed bacterial MV, and 12 of them (60%) had MDR, which could be treated with an intraventricular administration of colistin, tygecycline or amikacin. Of the 48 patients, 44 (91.6%) received intravenous (IV) antimicrobial treatment, and 12 (25%) received IV and intraventricular antimicrobial treatment via EVD catheters. The baseline characteristics of the patients are presented in Tables I, II and III. In the baseline demographic and pathophysiological characteristics of the patients, the parameters, incidence of diabetes mellitus and the sum of EVDs changed (Tables I and II), exhibited statistically significant differences between the groups (P<0.05). However, following univariate analysis for the survivors and non-survivors, the same parameters did not exhibit any statistically significant difference (Table IV).

In 12 out of the 20 (60%) patients in group B, the isolated microorganism in the CSF was *Acinetobacter baumannii*; in 6 patients (30%), *Klebsiella pneumoniae* was detected; and in 1 patient (5%), *Staphylococcus haemolyticus* was detected (Table III).

### Study outcomes

The clinical outcomes of the patients are presented in Table V. The durations of hospital stay and ICU stay were significantly lower in group A (32.4±24 and 21.1±11 days, respectively) compared to group B (54.7±37 and 42±24 days, respectively) (P=0.027 and P=0.001, respectively). The characteristics of the univariate analysis for survivors and non-survivors are presented in Table IV. The APACHE II score was significantly lower, and the EVD distance from the wound exit side to the burr hole was significantly shorter in the survivors compared to the non-survivors (17.5±6 and 15.4±4 mm vs. 22.5±6 and 39.8±38 mm, respectively) (Table IV).

**Figure 1:**
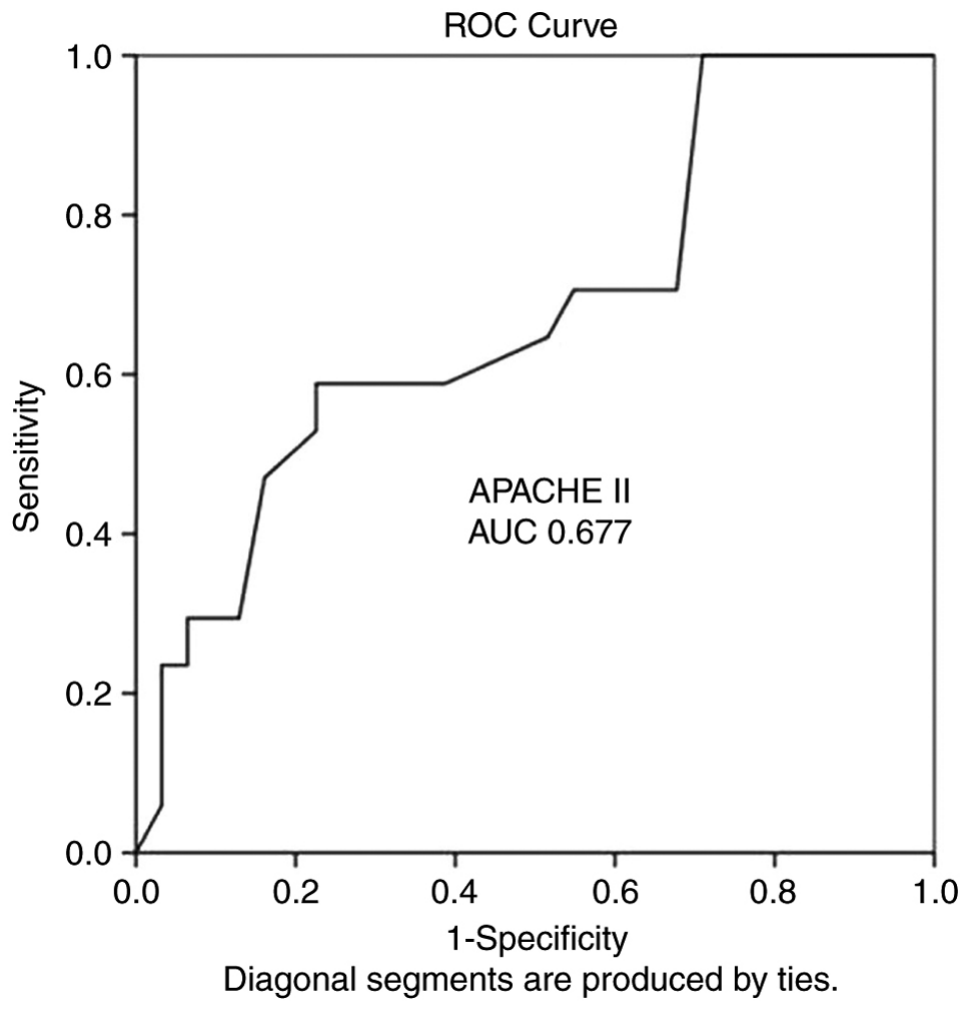
ROC analysis revealed of the APACHE II score. The results revealed that an AUC of 0.677, standard error of 0.082 (P=0.044) and a 95% confidence interval of 0.516-0.839), and a cut-off of 14 could predict poor outcomes (mortality) with a sensitivity of 100% and specificity of 71%. ROC, receiver operating characteristic; AUC, area under the ROC curve; APACHE II, Acute Physiological and Chronic Health Evaluation II.

**Figure 2:**
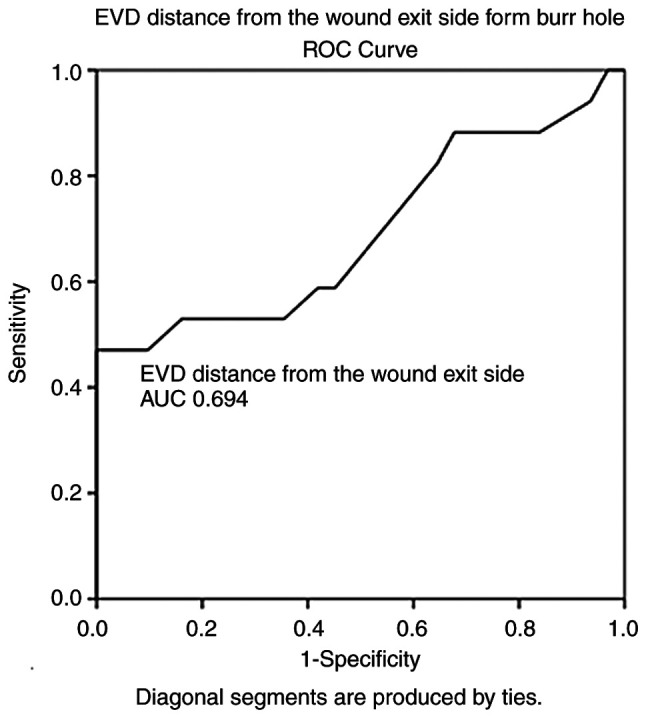
ROC analysis of EVD distance from the wound exit side from the burr hole. The results revealed that an AUC of 0.694 and standard error of 0.088 (P=0.028) and a 95% confidence interval of 0.521-0.866, and a cut-off of 11.5 mm could predict poor outcomes (mortality) with a sensitivity of 88% and specificity of 83%. ROC, receiver operating characteristic; AUC, area under the ROC curve; EVD, external ventricular drain.

ROC analysis demonstrated that the durations of hospital stay [AUC, 0.822; standard error (SE), 0.760; 95% confidence interval (CI), 0.673-0.970; P<0.05], ICU stay (AUC, 0.737; SE, 0.079; 95% CI, 0.583-0.892; P<0.05) and GCSexit (AUC, 0.968; SE, 0.026; 95% CI, 0.916-1.000; P<0.05) could predict poor outcomes (Table VI). Notably, ROC analysis also revealed that the APACHE II score (AUC, 0.677; SE, 0.082; 95% CI, 0.516-0.839; P=0.044) and with a cut-off value of 14 could predict poor outcomes (mortality) with a sensitivity of 100% and a specificity of 71%. In addition, the EVD distance from the wound exit side from the burr hole (AUC, 0.694; SE, 0.088; 95% CI, 0.521-0.866; P=0.028) and with a cut-off of 11.5 mm could predict poor outcomes (mortality) with a sensitivity of 88% and a specificity of 83% (Figs. 1 and 2, and Table VI).

The cure rates did differ not significantly between the groups: 18 out of 28 (64.3%) vs. 13 out of 20 (65%) (P=0.951). The GCS exit was 9.2±5 in group A and 9.4±5 in group B (P=0.885) (Table V).

## Discussion

The findings of the present study suggest that in patients receiving therapy with EVD for developed hydrocephalus, the severity of the clinical condition according to the APACHE II score was associated with survival. In addition, it was found that the EVD-related distance from the wound exit side to the burr hole could predict poor outcomes (mortality) in these patients. Notably, EVD duration over a mean of ≥17 days does not affect the outcomes of patients. Furthermore, changing EVDs between 9 and 12 days is a safe procedure without any risk for the development of MV. As regards the pathophysiological characteristics of the patients (Table II), the mean duration of EVD change was 17.6 days for all patients, 10.8 days for group A and 27.1 days for group B; the difference between the groups was statistically significant. Thus, the EVD duration, with a mean time of ≥17 days, did not affect the outcomes of patients. However, the mean duration of EVD change for survivors and non-survivors was 8.6±7 and 8.7±5 days and the different was not statistically significant (Table IV). Thus, a mean change of EVD between 9 and 12 days is a safe procedure without any risk for the development of MV and an EVD change after >12 or (8.7±5) days is critical for avoiding drain-related infections in the CNS.

EVD-related infections may arise from catheter colonization by the insertion of skin flora during placement (11). In the present study, the EVD distance from the wound exit side to the burr hole was related to poor outcomes. Conversely, a distance >11.5 mm could predict a better outcome.

Previous studies have reported various lengths of hospital stays in patients with MV following EVD placement, with a mean duration of 42-128 days (12-14). Post-surgical intracranial infection is a severe complication secondary to neurosurgical procedures, usually defined as bacterial (MV) following neurosurgery. It is main cause of prolonged hospital stay, increased medical costs, severe neurological dysfunction and even mortality (1). Although the incidence of MV is not high, the case fatality rate is usually >20% once MV occurs due to the special anatomical and physiological structure of the CNS. Effectively reducing the risk factors of MV and early identification and treatment is of utmost clinical significance to reduce the incidence and mortality rates associated with post-operative MV and to improve the prognosis of patients (1). In the present study, the duration of hospital stay was 32.4±24 days, which was shorter than that in the existing literature (1,12-14).

Furthermore, previous studies have reported that a longer drainage duration does not represent an increased risk of developing MV infection (15-17). On the other hand, other studies have reported that the risk of becoming infected with MV is significantly lower after day 9 of EVD (1,18).

Of note, other studies have concluded that a longer drainage duration affects the chances of infection (17,19). However, in the present study, the mean EVD duration was 17.6±12 days (10.8±5 days for those who had not developed MV and 27.1±14 days for patients with MV), and there was no association with poor outcomes. This may indicate that EVD duration, with a mean time of ≥17 days, does not affect the outcomes of patients. In addition, for the periods mentioned above, the sum of EVDs change was 1.6±1 in the total sample (1.2±0.6 and 2.3±1 for the patients without MV and those with MV, respectively). Thus, an EVD changed between 9 and 12 days is safe and does not pose any risk for the development of MV.

Studies have reported that the APACHE-II scoring system is useful for classifying patients according to their disease severity. Furthermore, a previous study demonstrated a negative relationship between a high score and the length of the hospital stay (20). In the present study, the value of the APACHE II score was determined as a mean score of 17.5±6 and was associated with an increased survival.

The present study had some limitations which should be mentioned. The main limitation was that it was conducted in a single center, and its retrospective nature was associated with possible errors in collecting and interpreting the data from the clinical history.

In conclusion, the present study determined the value of the APACHE II score and its association with an increased survival in patients treated with EVD. In addition, it was found that the EVD-related distance from the wound exit side of the burr hole could predict poor outcomes in these patients. Notably, EVD duration, with a mean time of ≥17 days, did not affect the outcomes of patients. As a result, avoiding an EVD change after >12 days is critical for avoiding drain-related infections in the CNS.

## Figures and Tables

**Table I:** Baseline demographic characteristics of the patients.

Parameter	All patients, n=48 (100%)	Group A, n=28 (58.3%)	Group B, n=20 (41.7%)	P-value
Age, mean ± SD (years)	55.2±17	47.3±16	60.8±17	<0.05
Sex (male), n (%)	32 (66.6)	17 (35.4)	15 (31.2)	0.301
Cause of admission				
ICH + or IVT, n (%) aSAH, n (%)	26 (54.1)	14 (29.1)	12(25)	0.363
Anterior circulation aneurysm, n (%)	14 (29.1)	8 (16.6)	6 (12.5)	
Posterior circulation aneurysm, n (%)	4 (8.3)	2 (4.1)	2 (4.1)	
TBI, n (%)	4 (8.3)	4 (8.3)	0 (0)	
Diabetes mellitus, n (%)	9 (18.7)	8 (16.6)	1 (2.0)	<0.05
Hypertension, n (%)	31 (64.5)	20 (41.6)	11 (22.9)	0.241
Hyperlipidemia, n (%)	15 (31.2)	11 (22.9)	4 (8.3)	0.155
GCS of admission, mean ± SD	7.4±3	7.6±3	7.2±2	0.735
APACHE II score, mean ± SD	19.3±7	19.9±7	18.4±6	0.745
Hemodialysis, n (%)	10 (20.8)	5 (10.4)	5 (10.4)	0.548
EVD, n (%)	35 (72.9)	20 (41.6)	15 (31.2)	0.784
Distance from the wound exit side to the burr hole, mean ± SD (mm)	24.1±25	27.2±32	19.7±10	0.494
Duration, mean ± SD (days)	8.6±6	7.5±6	10.2±7	0.193
DC, n (%)	18 (37.5)	10 (20.8)	8 (16.6)	0.762

ICH, intracranial hemorrhage; IVT, intraventricular hemorrhage; SAH, subarachnoid hemorrhage; TBI, traumatic brain injury; GCS, Glasgow Coma Scale; APACHE, Acute Physiologic Assessment and Chronic Health Evaluation; SD, standard deviation; DC, decompressive craniectomy.

**Table II:** Pathophysiological characteristics of the patients.

Parameter	All patients, n=48 (100%)	Group A, n=28 (58.3%)	Group B, n=20 (41.7%)	P-value
Fever of 10 days duration before MV, n (%)	12(25)	1 (2.0)	11 (22.9)	<0.05
CSF leak, n (%)	5 (10.4)	2 (4.1)	3 (6.2)	0.380
Sepsis, n (%)	27 (56.2)	8 (16.6)	19 (39.5)	<0.05
VAP, n (%)	27 (56.2)	13 (27.0)	14	0.105
UTI, n (%)	13 (27.0)	5 (10.4)	8 (16.6)	0.089
EVD				
Sum of EVDs changed, mean ± SD	1.6±1	1.2±0.6	2.3±1	<0.05
Duration, mean ± SD (days)	17.6±12	10.8±5	27.1±14	<0.05
IVentrT, n (%)	12(25)	0(0)	12(25)	<0.05
IVentrT duration of colistin, mean ± SD (days)	4.0±8	0.0±0	9.7±10	<0.05
IVentrT duration of tygecycline, mean ± SD (days)	2.2±5	0.0±0	5.4±8	<0.05
IventrT duration of amikacin, mean ± SD (days)	0.4±3	0.0±0	1.0±4	0.237
Intravenous antimicrobial treatment				
Colistin, n (%)	18 (37.5)	1 (2.0)	17 (35.4)	<0.05
Meropenem, n (%)	4 (8.3)	0 (0)	4 (8.3)	<0.05
Amikacin, n (%)	1 (2.0)	0 (0)	1 (2.0)	0.232
Linezold, n (%)	1 (2.0)	0 (0)	1 (2.0)	0.232
Tygecycline, n (%)	11 (22.9)	1 (2.0)	10 (20.8)	<0.05
Ceftazidime/avibactam, n (%)	2 (4.1)	0 (0)	2 (4.1)	0.087
Trimethoprim/sulfamethoxazole, n (%)	1 (2.0)	0 (0)	1 (2.0)	0.232
Ampicillin/sulbactam n (%)	5 (10.4)	0 (0)	5 (10.4)	<0.05
Gentamicin, n (%)	1 (2.0)	0 (0)	1 (2.0)	0.232

CSF, cerebral spinal fluid; VAP, ventilator-associated pneumonia; UTI, urinary tract infection; EVD, external ventricular drain; IVentrT, intraventricular treatment; SD, standard deviation.

**Table III:** Isolated microorganisms from CSF of patients with EVD-related infections.

Microorganism	All 20 (100%)
*Acinetobacter baumanii*, n (%)	12(60)
*Klebsiella pneumoniae*, n (%)	6(30)
*Staphylococcus haemolyticus*, n (%)	1(5)
Polymicrobial (*Acinetobacter baumanii and Enterobacter Enterobacter cloacae*), n (%)	1(5)

CSF, cerebral spinal fluid; EVD, external ventricular drainage.

**Table IV:** Univariate analysis for survivors and non-survivors.

Parameter	Survivors, n=31 (64.6%)	Non-survivors, n=17 (35.4%)	P-value
Group A, n (%)	18 (64.3)	10 (35.7)	0.951
Group B, n (%)	13(65)	7(35)	
Age, mean ±SD (years)	54.2±19	55.7±31	0.829
Sex (male), n (%)	21 (43.7)	11 (22.9)	0.301
Cause of admission			
ICH + or IVT, n (%)	17(35.4)	9(18.7)	0.792
aSAH, n (%)			
Anterior circulation aneurysm, n (%)	10 (20.8)	4 (8.3)	
Posterior circulation aneurysm, n (%)	2 (4.1)	2 (4.1)	
TBI, n (%)	2 (4.1)	2 (4.1)	
Diabetes mellitus, n (%)	5 (10.4)	4 (8.3)	0.530
Hypertension, n (%)	18 (37.5)	13 (27.0)	0.202
Hyperlipidemia, n (%)	7 (14.5)	8 (16.6)	0.080
GCS of admission, mean ± SD	7.9±3	6.5±2	0.174
APACHE II score, mean ± SD	17.5±6	22.5±6	<0.05
Hemodialysis, n (%)	4 (8.3)	6 (12.5)	0.068
EVD, n (%)	21 (43.7)	14 (29.1)	0.276
Distance from the wound exit side to the burr hole, mean ± SD (mm)	15.4±4	39.8±38	<0.05
Duration, mean ± SD (days)	8.6±7	8.7±5	0.974
DC, n (%)	12(25)	6 (12.5)	0.815
Fever of 10 days duration before MV, n (%)	8 (16.6)	4 (8.3)	0.862
CSF leak, n (%)	3 (6.2)	2 (4.1)	0.821
Sepsis, n (%)	19 (39.5)	8 (16.6)	0.342
VAP, n (%)	17 (35.4)	10 (20.8)	0.790
UTI, n (%)	9 (18.7)	4 (8.3)	0.682
EVD			
Sum of EVDs changed, mean ± SD	1.6±0.9	1.8±1	0.742
Duration, mean ± SD (days)	17.5±11	17.7±15	0.503
IVentrT, n (%)	7 (14.5)	5 (10.4)	0.601
IVentrT duration of colistin, mean ± SD (days)	4.3±8	3.5±8	0.832
IVentrT duration of tygecycline, mean ± SD (days)	1.4±4	3.6±7	0.200
IventrT duration of amikacin, mean ± SD (days)	0.0±0	1.2±5	0.177
Intravenous antimicrobial treatment			
Colistin, n (%)	12(25)	6 (12.5)	0.815
Meropenem, n (%)	2 (4.1)	2 (4.1)	0.524
Amikacin, n (%)	0 (0)	1 (2.0)	0.172
Linezold, n (%)	1 (2.0)	0 (0)	0.454
Tygecycline, n (%)	7 (14.5)	4 (8.3)	0.940
Ceftazidime/avibactam, n (%)	1 (2.0)	1 (2.0)	0.660
Trimethoprim/sulfamethoxazole, n (%)	0 (0)	1 (2.0)	0.172
Ampicillin/sulbactam n (%)	3 (6.2)	2 (4.1)	0.821
Gentamicin, n (%)	1 (2.0)	0 (0)	0.454
Hospital stay, mean ± SD (days)	49.7±32	27.2±27	<0.05
ICU stay, mean ± SD (days)	34.4±21	21.4±16	<0.05

ICH, intracranial hemorrhage; IVT, intraventricular hemorrhage; SAH, subarachnoid hemorrhage; TBI, traumatic brain injury; GCS, Glasgow Coma Scale; APACHE, Acute Physiologic Assessment and Chronic Health Evaluation; SD, standard deviation; DC, decompressive craniectomy; CSF, cerebrospinal fluid; VAP, ventilator-associated pneumonia; UTI, urinary tract infection; EVD, external ventricular drain; IVentrT, intraventricular treatment; SD, standard deviation; ICU, intensive care unit.

**Table V:** Outcomes of the patients with external ventricular drainage.

Parameter	All patients, n=48 (100%)	Group A n=28	Group B n=20	P-value
Mortality, n (%)	17 (35.4)	10 (35.7)	7(35)	0.959
Hospital stay, mean ± SD (days)	41.7±32	32.4±24	54.7±37	<0.027
ICU stay, mean ± SD (days)	29.8±20	21.1±11	42±24	<0.001
GCSexit	9.3±5	9.2±5	9.4±5	0.885
Cured, n (%)	31 (64.5)	18 (64.3)	13(65)	0.959

SE, standard error; ICU, intensive care unit; GCSexit, Glasgow Coma Scale in patients who were discharged alive.

**Table VI:** ROC analysis (outcome: Mortality).

Parameter	AUC	SE	95% CI, lower-upper	P-value
APACHE II score	0.677	0.082	0.516-0.839	<0.0044
EVD; distance from the wound exit side to the burr hole	0.694	0.088	0.521-0.866	<0.028
Duration of hospital stay	0.822	0.760	0.673-0.970	<0.05
Duration of ICU stay	0.737	0.079	0.583-0.892	<0.05
GCSexit	0.968	0.026	0.916-1.000	<0.05

APACHE, Acute Physiologic Assessment and Chronic Health Evaluation; ICP, intracranial pressure; ICU, intensive care unit; GCSexit, Glasgow Coma Scale in patients who were discharged alive; AUC, area under the curve; CI, confidence interval; SE, standard error.

## Data Availability

The datasets used and/or analyzed during the current study are available from the corresponding author on reasonable request.
